# Surface Micro-Patterned Biofunctionalized Hydrogel for Direct Nucleic Acid Hybridization Detection

**DOI:** 10.3390/bios13030312

**Published:** 2023-02-23

**Authors:** Paola Zezza, María Isabel Lucío, Estrella Fernández, Ángel Maquieira, María-José Bañuls

**Affiliations:** 1 Instituto Interuniversitario de Investigación de Reconocimiento Molecular y Desarrollo Tecnológico (IDM), Universitat Politécnica de Valéncia, Universitat de Valéncia, Camino de Vera s/n, 46022 Valencia, Spain; 2 Departamento de Química, Universitat Politécnica de Valéncia, Camino de Vera s/n, 46022 Valencia, Spain

**Keywords:** hydrogels, surface micropattern, probe immobilization, photoclick reaction, diffraction, label-free

## Abstract

The present research is focused on the development of a biofunctionalized hydrogel with a surface diffractive micropattern as a label-free biosensing platform. The biosensors described in this paper were fabricated with a holographic recording of polyethylene terephthalate (PET) surface micro-structures, which were then transferred into a hydrogel material. Acrylamide-based hydrogels were obtained with free radical polymerization, and propargyl acrylate was added as a comonomer, which allowed for covalent immobilization of thiolated oligonucleotide probes into the hydrogel network, via thiol-yne photoclick chemistry. The comonomer was shown to significantly contribute to the immobilization of the probes based on fluorescence imaging. Two different immobilization approaches were demonstrated: during or after hydrogel synthesis. The second approach showed better loading capacity of the bioreceptor groups. Diffraction efficiency measurements of hydrogel gratings at 532 nm showed a selective response reaching a limit of detection in the complementary DNA strand of 2.47 µM. The label-free biosensor as designed could significantly contribute to direct and accurate analysis in medical diagnosis as it is cheap, easy to fabricate, and works without the need for further reagents.

## 1. Introduction

Nowadays, the interest in developing affordable and mass-producible clinical diagnostics devices is increasing to improve accessibility to healthcare worldwide. Having fast and self-monitoring tests that allow detection onsite is a global interest to avoid hospital crowding and the spreading of contagious diseases. Definitely, the development of portable devices for point-of-care testing (POCT), which allows fast analyte detection with an easily interpretable readout, is crucial for the future [1]. POCT is presently available for a variety of analyses, for example, pregnancy tests, infectious disease tests (such as respiratory infections and sexually transmitted diseases), glucose tests, and several other applications [2,3,4,5,6]. Among various types of sensors, optical biosensors present great advantages over conventional analytical techniques because they enable direct, real-time, and label-free detection of many biological and chemical substances [7,8,9]. Their advantages include high sensitivity, small size, light weight, cost-effectiveness, and the ability to provide multiplexed or distributed sensing. In this context, holographic biosensors offer an appealing approach for label-free optical biosensing. Holographic sensors are gratings, recorded with holographic techniques, of functionalized polymers capable of quantifying the concentration of the target analyte [10]. As a transducer, a holographic pattern is recorded in the sensitive polymer structure, which consists of a 3D periodic structure with alternating strips of differing refractive index (RI), and thus it diffracts the light. After the holographic recording, the polymer matrix, permeable to the target analyte, changes its physical and chemical characteristics, such as lattice spacing and/or refractive index based on its interaction with the target analyte, and produces a change in the diffraction pattern. So far, various hydrophilic and hydrophobic polymers have been used for the fabrication of holographic sensors including gelatin, poly(2-hydroxyethyl methacrylate) (pHEMA), poly(acrylamide) (pAAM), and polyvinyl alcohol (PVA). Their application includes humidity, temperature, and pressure sensors, as well as glucose, lactate, electrolytes, and pH chemical sensors [11]. However, there are very few examples using bioreceptors, mainly antibodies, to achieve holographic biosensing, with their use for nucleic acid hybridizations not being reported. In this work, an Acrylamide/Propargyl acrylate (AM/PA) hydrogel is used, simultaneously, as a matrix for the holographic pattern fabrication and for the functionalization with single-strand thiolated oligonucleotides as a biorecognition element. Hydrogels are attractive platforms for bioanalysis thanks to their ability to retain large amounts of water, acting like biological tissues, optimal for biological interactions [12,13,14,15,16]. Hydrogel-based sensors found numerous applications in clinical diagnostics, biomedical research, environmental monitoring, and food testing [17,18,19,20,21]. Thus, because of their properties, hydrogels have been employed in POC systems for different purposes, which include cell and tissue immunostaining [22], localized photothermal heating [23], microneedle fabrication for drug delivery [24] or for interstitial fluid sampling [25], ion sensing [20], and cocaine, ochratoxin A [26], and glucose detection [27] as well as mRNA detection with hydrogel microparticles [28].

Here, a rapid, specific, and label-free detection system for nucleic acid hybridization based on surface relief holographic gratings was demonstrated. To this aim, the surface of an oligonucleotide probe-functionalized hydrogel was micro-patterned [29]. Briefly, Acrylamide/Propargyl acrylate (AM/PA) hydrogels were obtained using the free radical polymerization (FRP) reaction, both thermally and photochemically activated. Using replica molding of holographic molds [30], a diffractive micropattern on the hydrogel surface was fabricated. It acts as a transducer that diffracts light, producing a measurable signal proportional to the probe–target interaction. The surface micropatterning technique that was used has some advantages: it is easy to manufacture, does not require expensive instrumentation, and allows the creation of patterns of micrometer size. To apply this surface micropatterned hydrogel in biosensing, DNA probes were incorporated into the network as bioreceptors for the target. In particular, covalent functionalization of thiol-modified ssDNA probes in acrylamide-based hydrogels was obtained using a photoclick thiol-ene reaction [31]. Hence, when hybridizing with the complementary strand, the hydrogel underwent changes that were monitored with optical diffraction measurements. The change in the diffraction efficiency of hydrogel gratings was specific for the complementary strand, given that this is the first time that holographic hydrogel gratings are used to detect the direct hybridization of oligonucleotides.

## 2. Materials and Methods

### 2.1. Chemicals

Acrylamide (AM), propargyl acrylate (PA), N, N′-methylenebis (acrylamide) (MBA), Potassium persulfate (KPS), 2,2-Dimethoxy-2-phenylacetophenone (DMPA) and Tetrahydrofuran (THF), sodium phosphate dibasic, potassium phosphate monobasic, sodium chloride, potassium chloride, sodium acetate, sodium citrate, ethylenediaminetetraacetic acid, and Tween-20 were purchased from Sigma–Aldrich (Madrid, Spain). The acetate-Tris (2-carboxyethyl) phosphine buffer (Ac-TCEP, pH 4.5) consists of 25 mM of TCEP, 0.15 M sodium acetate, 0.1 M Ethylenediaminetetraacetic acid, and 0.1 M NaCl in DI water; the phosphate-buffered saline solution with 0.1% (*v*/*v*) of Tween 20 detergent (PBS-T, pH 7.4) consists of 137 mM NaCl, 2.7 mM KCl, 10 mM Na_2_HPO_4_, 1.8 mM KH_2_PO_4_; and the saline-sodium citrate buffer (SSC1x, pH 7.4) consists of 0.15 M NaCl and 0.015 M sodium citrate. Polydimethylsiloxane (PDMS) Sylgard 184 was purchased from Dow Corning (Wiesbaden, Germany). The oligonucleotides were supplied by Sumilab (Valencia, Spain), and the sequences used are listed in Table S1.

### 2.2. Equipment

Hydrogel photopolymerization and bioreceptor immobilization with UV irradiation was carried out using a UV photoreactor LightOx PhotoReact 365 nm (13 mW/cm^2^ light power) (Sigma–Aldrich, Madrid, Spain). Hydrogel fluorescence measurements were registered with a fluorescence microarray analyzer SensoSpot (Miltenyi Imaging GmbH, Radolfzell, Germany) (λ_ex_ = 633 nm, λem = 670 nm). Fluorescence image data processing was performed with the GenePix Pro 4.0 software from Molecular Devices, Inc. (Sunnyvale, CA, USA).

The morphological characterization of hydrogels was carried out using scanning electron microscopy (SEM, Gemini SEM 500 system, Zeiss, Oxford Instruments, Oxford, UK). Hydrogels were completely swollen in distilled water and frozen at −20 °C. Then, they were lyophilized overnight (Telstar Lyoquest freeze-drier, Azbil Telstar Technologies, S. L. U., Terrasa, Spain) to yield completely dry aerogel samples. Finally, dry samples were prepared using sputter coating with a Au layer of about 15 nm (BAL-TEC SCD 005 sputter coater, Leica microsystems, Wetzlar, Germany).

Fourier transform infrared (FT-IR) spectroscopy of lyophilized hydrogels was performed using a Tensor 27 FT-IR-spectrophotometer (Bruker, MA, USA). UV-Visible spectra of hydrogels immersed in H_2_O were collected in an Agilent 8453 spectrophotometer (Santa Clara, CA, USA). For the analysis, hydrogels were polymerized inside an Eppendorf and, after washing, they were placed inside a 1 × 1 cm cuvette filled with H_2_O.

Swelling behavior studies were carried out with lyophilized hydrogel samples. Samples with a size of approximately 1 cm^3^ were immersed in PBS-T (10 mL) at room temperature. The weight of the swollen hydrogels was recorded at different times until they were totally swollen (reaching a constant weight). Buffer excess on the surface of the hydrogel was removed with filter paper before weighing. The swelling degree was calculated using Equation (1), where Wt is the weight of the hydrogel after being immersed in the buffer during time “t” and W0 is the weight of the lyophilized hydrogel before the immersion.

(1) $$\mathrm{Swelling}\;(\%)=\frac{\mathrm{Wt}-W0}{\mathrm{Wo}}\;\times 100$$

### 2.3. Hydrogel Synthesis

Acrylamide/Propargyl acrylate (AM/PA) and acrylamide (AM) hydrogels were prepared using free radical polymerization (FRP) either with photochemical or thermal activation (Scheme S1). Different hydrogel compositions were optimized: AM(25)/PA, AM(8)/PA, AM(25), and AM(8). The AM(25)/PA hydrogel was prepared by mixing 25% (*w*/*v*) of AM monomer, 0.05% (*w*/*v*) of MBA crosslinker, and 15 μL of PA co-monomer in 1 mL of distilled water. The AM(8)/PA hydrogel was prepared by mixing 8% (*w*/*v*) of AM monomer, 0.25% (*w*/*v*) of MBA crosslinker, and 15 μL of PA co-monomer in 1 mL of distilled water. The control hydrogel AM(25) was prepared by mixing 25% (*w*/*v*) of AM monomer and 0.05% (*w*/*v*) of MBA crosslinker, while the control hydrogel AM(8) was prepared by mixing 8% (*w*/*v*) of AM monomer and 0.25% (*w*/*v*) of MBA crosslinker. For the synthesis of the hydrogel using thermal activation, potassium persulfate (KPS) at 1% (*v*/*v*) was added to the solution as a thermal initiator, and the reaction mixtures were placed in an oven at 60 °C for 90 min. For the synthesis of hydrogels with photochemical activation, 2,2-Dimethoxy-2-phenylacetophenone (DMPA) photoinitiator at 1% (*w*/*v*) was added to the reaction mixture and hydrogels were polymerized irradiating at 365 nm in a UV photoreactor (13 mW/cm^2^) for 10 min. Once polymerized, the hydrogels were washed with immersion in distilled water for at least 2 h using three times fresh water to ensure that non-polymerized monomers were eliminated. The obtained hydrogels were stored completely swollen in distilled water at 4 °C.

### 2.4. Probe Immobilization and Hybridization Assay

For potential biosensing applications, AM/PA hydrogels and their control systems, AM hydrogels, were covalently functionalized with a thiol-modified oligonucleotide probe, and hybridization capacity was tested with a fluorescence-labeled target. All probes used are listed in Table S1. The bioreceptor immobilization was studied either during or after the hydrogel synthesis. In the first approach, after monomers and crosslinker homogenization in water, 1 μM of Probe 1 and 1% (*w*/*v*) of DMPA photoinitiator in water were added to the mixture, and the solution was irradiated at 365 nm (13 mW/cm^2^) for 10 min. In this strategy, polymerization and bioreceptor immobilization were carried out simultaneously in one step. In the second approach, the already thermally synthesized hydrogels were cut into squares (0.5 × 0.5 cm) and immersed in 100 µL of 1 μM of Probe 1 and 1% (*w*/*v*) of DMPA photoinitiator in THF:Ac-TCEP 1:1. Then, the hydrogels were irradiated at 365 nm (13 mW/cm^2^) for 30 min. In both approaches, after the immobilization step, the hydrogels were placed on an oscillator plate and washed overnight with PBS-T.

For the hybridization assays, Probe 1-functionalized hydrogels of 0.5 × 0.5 cm were placed in a transparent ELISA (enzyme-linked immunosorbent assay) plate and equilibrated in 250 µL of SSC1x for 24 h. Then, SSC1x was discarded, and the hydrogels were incubated with 50 µL of Cy5-labeled, complementary strand Target 2, in SSC1x, at growing concentrations (0; 0.2; 0.4; 0.8; 1; 1.5; and 2 µM) for one hour at 37 °C. Fluorescence signals were collected immediately after the hybridization and after overnight washing with SSC1x. Control hydrogels having immobilized a non-complementary sequence (Probe 2) were also hybridized as described.

### 2.5. Surface Micropattern Fabrication

Surface microstructures made of Polyethylene terephthalate (PET) were fabricated using the direct laser interference patterning (DLIP) technique [32]. The DLIP system was equipped with a frequency quadrupled Q-switched laser head (TECH-263 Advanced Laser-export Co., Ltd., Moscow, Russia) with a maximum pulse energy of 50 μJ, operating at a wavelength of 263 nm and with a pulse duration shorter than 3 ns. A fluence of 0.09 J/cm^2^ was used to obtain PET masters with a period of approximately 4 μm. The structural features of the original PET master were characterized with a 3D optical profilometer (Sensofar, PLu neon, Terrasa, Spain). Hydrogel surface micropatterns were fabricated using the replica molding technique (REM) from the original PET master. The micro-pattern obtained on the hydrogel surface was observed with optical microscopy (OM, Leica microsystems, MZ APO, Wetzlar, Germany).

**Scheme 1:**
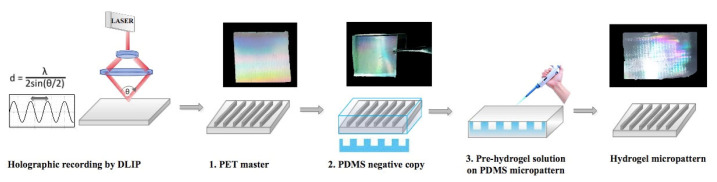
Micropatterning process steps for hydrogel surface structure manufacturing.

Micropatterns were obtained in the hydrogel surface using replica molding (Scheme 1). Firstly, the original PET micropattern was copied onto PDMS. The PDMS solution was poured onto the PET surface, a vacuum was applied for 10 min to aid the solution-pattern adhesion, and then it was placed in the oven at 60 °C for 2 h. Secondly, the PDMS negative pattern was transferred onto the hydrogel surface. Initially, pre-polymeric solutions with monomers and crosslinkers of hydrogels AM(25)/PA, AM(25), AM(8)/PA, and AM(8) were stirred for 20 min until homogenization. Then, KPS was added, and the solution was sonicated for 2 min. The solutions were poured onto different PDMS micropatterned surfaces, a vacuum was applied for 10 min, and then they were placed in an oven at 60 °C for 1.5 h. Once polymerized, they were peeled off and washed with immersion in distilled water for at least 2 h using three times fresh water to ensure that non-polymerized monomers were eliminated. The micropatterned hydrogels were stored completely swollen in distilled water at 4 °C.

**Figure 1:**
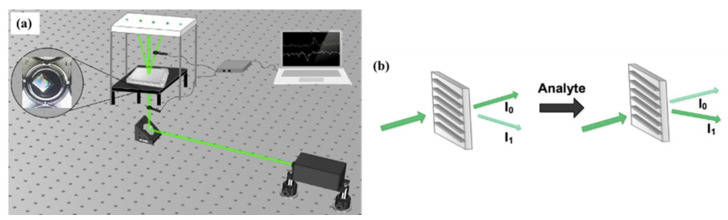
(**a**) Optical set-up used for diffraction efficiency measurement. (**b**) Analyte sensing principle: after analyte biorecognition, the intensity of zero and first diffraction order changes, and thus the diffraction efficiency.

The micropatterns obtained on the PDMS and the swollen hydrogel surface were observed with optical microscopy (OM, Leica microsystems, MZ APO, Wetzlar, Germany). Surface pattern characterization was also carried out with an optical set-up as shown in Figure 1. From the bottom, a continuous green laser beam (532 nm, 100 mW) is attenuated and orthogonally directed to the sample holder using a mirror. The sample holder is a 3D-printed platform provided with a pinhole and patterned lanes that allow the x–y movement of a 96-well ELISA plate so the laser beam can be unequivocally directed toward every well. Then, movable silicon photodiodes are placed after the sample holder to record the intensity of the different laser beams (incident or diffracted). A concave spherical lens (f = 30 mm) was placed on the top of the 96-well plate to focus the diffracted beams produced by the hydrogel micropatterns.

Diffraction efficiency (DE%) of the micropatterns was calculated with Equation (2):

(2) $$\mathrm{DE}\;(\%)=\frac{I_{1}}{I_{0}}\;\times \;100$$

where I_0_ was the intensity of the zero-diffraction order and I_1_ was the intensity of the first diffracted order.

### 2.6. Label-Free Hybridization Assay

Bioreceptor immobilization in micropatterned hydrogels was carried out in two steps. Firstly, thermally polymerized micropatterned hydrogel (AM(25)/PA) was functionalized with 5 µM of Probe 1. For that, micropatterned hydrogels were cut in squares (0.5 × 0.5cm) and treated with 100 µL of a 5 μM solution of Probe 1 and 1% (*w*/*v*) of DMPA photoinitiator in THF:Ac-TCEP 1:1. Then, the hydrogels were irradiated at 365 nm (13 mW/cm^2^) for 30 min. The functionalized micropatterned hydrogels were washed overnight with PBS-T to eliminate the non-covalently attached probes. For the label-free hybridization assays, the probe-functionalized micropatterned hydrogels were placed in separated wells of a transparent ELISA plate and equilibrated in 250 µL of SSC1x. The day after, SSC1x buffer solution was replaced with a fresh one and the initial diffraction efficiencies (DE_i_) of the hydrogels were obtained using the optical set-up (Figure 1) and Equation (2). Hybridization assay was performed using incubation of the hydrogels with growing concentrations of Target 1 (0; 2; 5; 10; and 25 µM) in 50 µL SSC1x for one hour at 37 °C. The hybridization experiment was also carried out with the AM(25)/PA hydrogel functionalized with a non-complementary, thiol-bearing oligonucleotide sequence (Probe 2), and hybridized at 10 and 25 µM of Target 1, as a negative control. Then, the hydrogels were washed overnight with SSC1x to be sure that all the non-specifically bound targets were removed. The final diffraction efficiencies of the hydrogels (DE_f_) were obtained using the optical set-up (Figure 1) and Equation (2). The relative diffraction efficiency was used to characterize the response of the hydrogel to the target concentration, as described in Equation (3):

(3) $$\mathrm{RDE}\left(\%\right)=\frac{{\mathrm{DE}}_{f}-{\mathrm{DE}}_{i}}{{\mathrm{DE}}_{i}}\times 100$$

where RDE is the relative diffraction efficiency, DE_i_ is the initial diffraction efficiency (after the equilibration step with SSC1x), and DE_f_ is the final diffraction efficiency (after incubation and washing steps) for the first diffraction order. All experiments were repeated three times.

## 3. Results and Discussion

### 3.1. Optimized Hydrogel Compositions

First, hydrogel composition was optimized from both a physical and a chemical point of view. Polyacrylamide hydrogels are one of the most utilized materials in the synthesis of holographic and photonic hydrogel due, among other things, to their excellent optical properties [11]. AM was chosen as the main monomer for the synthesis of the hydrogel networks and MBA as one of the most common crosslinkers for polyacrylamide. The PA co-monomer was incorporated to introduce the alkyne moiety, which was necessary for the further thiolated-probe covalent attachment through thiol-yne photo-click coupling chemistry [33]. Apart from reaching adequate physical and optical properties such as good porosity, transparency, and low optical background, the chemical formulation was adapted to increase the immobilization density of the biorecognition Probe 1. For that, different ratios of monomer (AM), co-monomer (PA), and crosslinker (MBA) were assayed. All the assay compositions are shown in Table S2. As expected, all the hydrogels were transparent with almost zero absorbance at the working wavelength of our system (532 nm). Figure S1 shows the UV-Visible spectra of all hydrogels. However, not all the synthesized hydrogels showed the consistency required for part of our purposes: the fabrication of surface relief diffraction grating using replica molding. The requirements of hydrogels for potentially yielding suitable gratings include the following: they must adapt the form of the container used for the polymerization and they need to be manipulable, easy to cut, not brittle, and to keep the macroscopical form after washing and swelling. The consistency of the different synthesized hydrogels polymerized with thermal activation is indicated in Table S2. In addition, Figure S2 shows photographs of hydrogels with different consistencies. 

AM(25)/PA and AM(8)/PA showed the best consistency and potential to be used as surface relief gratings for DNA hybridization, so they, and their counterpart controls without PA, were selected for further optimization. The selected compositions are shown in Table 1 and photographs of the hydrogels are shown in Figure S3. As the activation process for polymerization can affect the final properties of the hydrogel, i.e., porosity, swelling, etc., the polymerization was carried out following two different activation processes: thermally and photochemically.

**Figure 2:**
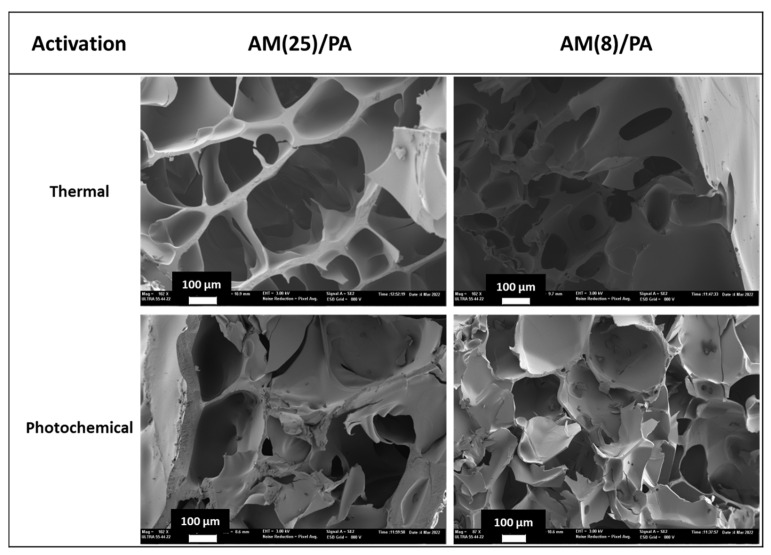
Porosity observed with SEM (scanning electron microscopy) for selected hydrogel compositions (AM(25)/PA) and (AM(8)/PA) prepared using thermal and photochemical activation.

The morphology of the optimized hydrogel compositions that contain PA was comparatively observed for thermal and photochemical activation, as poor homogeneity has been previously reported in hydrogels polymerized with UV-light [34,35,36]. For that, lyophilized hydrogels were analyzed with SEM (Figure 2 and Figure S4). As can be observed in the SEM micrographs, the thermal activation provided higher homogeneity and porosity to the hydrogel network for both the AM(25)/PA and AM(8)/PA compositions, although both activation procedures resulted in adequate porosity levels.

As the hydrogels obtained with thermal activation showed the best homogeneity based on the SEM, swelling behavior studies of these hydrogels were carried out to test the hydrogel buffer absorption capacity. In Figure S5 of the Supplementary Materials, the swelling studies show how the chemical composition affects the hydrogel water uptake. Hydrogels AM(8) demonstrated a higher swelling degree than hydrogels AM(25). This is probably because the larger quantity of monomer used in AM(25) hydrogels counteracts the higher crosslinker degree present in AM(8) hydrogels. Equally, the propargyl acrylate co-monomer contributed to the polymer swelling capacity. PA reduces the buffer absorption in AM(25)/PA and AM(8)/PA hydrogels in comparison to AM(25) and AM(8) reference systems, probably due to the higher hydrophobicity of the alkyne moiety. However, in both compositions, the swelling capacity was over 400%. Thus, the optimized compositions were tested for subsequent bioreceptor immobilization and surface micropatterning.

### 3.2. Probe Immobilization and Hybridization Assay

**Figure 3:**
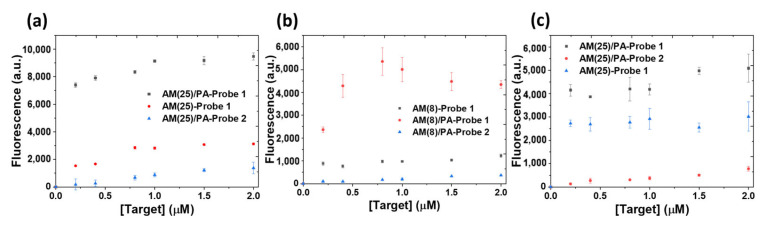
Fluorescence intensity measured after hybridization with increasing concentrations of labeled Target 2 (**a**) in AM(25)/PA and AM(25) hydrogels and (**b**) in AM(8)/PA and AM(8), biofunctionalized with Probe 1 or 2 after their polymerization; and (**c**) in AM(25)/PA and AM(25) hydrogels with Probe 1 or 2 covalently attached during the polymerization step. The Probe 1 sequence was complementary to Target 2, while the Probe 2 sequence was non-complementary; both probes bear the thiol moiety needed for thiol-yne or thiol-ene coupling. Details of the obtained fluorescence signals are shown in Section S-VI of the Supplementary Materials (Figures S8, S9, S10 and S11).

AM/PA hydrogels and their corresponding controls (without PA) were covalently functionalized with a thiol-bearing oligonucleotide probe for potential biosensing applications. The oligonucleotide probe acts as the specific biorecognition element for its complementary sequence (target). In the hydrogel formulation, the propargyl acrylate (PA) co-monomer had a C–C triple bond that was expected to enhance the binding with thiol-probes, in comparison to the control system [37]. Thiolated probes incorporation was carried out using the thiol-yne photoclick coupling reaction with UV irradiation at 365 nm (Scheme S2). Previous work by our group performed in microarray format had demonstrated that these irradiation conditions did not affect the probes stability and bioavailability to hybridize with the complementary strands [38]. Firstly, the thermally polymerized AM(25)/PA and AM(8)/PA hydrogels were biofunctionalized as the thermal activation yielded hydrogels with higher homogeneity and porosity, and, in addition, they showed a high swelling degree. Hydrogels were functionalized with Probe 1, complementary to the target, and, additionally, with Probe 2, which was a thiolated, non-complementary sequence. In addition, hydrogels without PA, AM(25) and AM(8), were also submitted to functionalization with Probe 1 to assess the role of PA in the probe immobilization process. The immobilization was carried out in 1:1 THF:Ac-TCEP, and TCEP was added to facilitate the reduction of disulfide bonds established between the thiolated probes. After probe immobilization, a fluorescence-labeled target sequence was used for hybridization assays to verify the successful incorporation of the thiol probe and its bioavailability for the specific hybridization. Therefore, thermally activated, probe-biofunctionalized hydrogels AM(25)/PA, AM(25), AM(8)/PA, and AM (8) were hybridized with increasing concentrations of the Cy5-labeled target sequence (Target 2) for 1h at 37 °C, and the fluorescence was registered after washing overnight (Figure 3a,b). As a control, a fluorescence signal was also registered after hybridization in several cross-section pieces of the hydrogels AM(8)/PA and AM(25)/PA to demonstrate that target 2 could reach the probe within 1h (Figure S7). Figure 3a,b show that significantly higher fluorescence signals (4-fold to 5-fold) were observed for AM(25)/PA and AM(8)/PA hydrogels compared to their control systems AM(25) and AM(8) when they were functionalized with Probe 1, complementary to the target. As expected, the introduction of the PA co-monomer allowed a much more effective probe immobilization, thanks to the thiol-yne coupling chemistry, increasing the probe loading in the hydrogels. Therefore, the immobilization strategy was successful for both AM(25)/PA and AM(8)/PA hydrogels. Moreover, a higher fluorescence signal was measured for the AM(25)/PA hydrogel in comparison to the AM(8)/PA hydrogel. In addition, almost no fluorescence was observed when AM(25)/PA and AM(8)/PA hydrogels were functionalized with Probe 2, having the non-complementary sequence, which demonstrated that specific hybridization was taking place, and non-specific binding was negligible inside the hydrogel supports. As polymerization could be also activated photochemically using the same wavelength needed for the thiol-yne coupling reaction, a second strategy was assessed for the hydrogels biofunctionalization: a one-step process that consisted of the immobilization of the thiolated probe during hydrogel polymerization. In this strategy, the thiol-yne photoclick coupling reaction and acrylamide polymerization, using DMPA as a photoinitiator, were triggered with UV irradiation at the same time. Therefore, pre-polymeric solutions of AM(25)/PA and AM(25) hydrogels were mixed with 1 μM of complementary Probe 1 and DMAP, and then irradiated at 365 nm for 30 min. Additionally, a control experiment was carried out with AM(25)/PA hydrogel and the non-complementary Probe 2. Once hydrogels were washed and equilibrated with SSC1x, hybridization assays with the Cy5-labeled target sequence (Target 2) at increasing concentrations, as above, were carried out, and fluorescence was registered after washing (Figure 3c). In this case, the highest fluorescence signal was also observed for hydrogels AM(25)/PA functionalized with Probe 1. However, the high fluorescence observed in the hybridization curve of hydrogel AM(25) showed that the thiolated probe resulted in being immobilized without the presence of (PA) co-monomer. This is due to the thiol-acrylate coupling reaction which follows the same principle as thiol-yne photocoupling reaction [39]. Figure S6 shows the IR spectrum of a lyophilized AM(25) hydrogel, which showed a spectral profile compatible with the presence of residual unreacted acrylamide groups. However, even in this case, the presence of PA increased the hydrogel probe immobilization capability. As before, AM(25)/PA hydrogels biofunctionalized with Probe 2 did not show a significant fluorescence signal after hybridization, which reveals that non-specific binding is also avoided with the one-pot functionalization strategy. Comparing the two strategies for AM(25)/PA hydrogels functionalized with Probe 1, complementary to Target 2, the ones biofunctionalized after polymerization (Figure 3a) showed two-fold the fluorescence signal of the ones biofunctionalized during the polymerization (Figure 3c). Probably, in the case of the biofunctionalization after the polymer synthesis, a larger number of bioreceptors are introduced and, in addition, these probes are more accessible to the target. Thus, thermally polymerized AM(25)/PA hydrogels biofunctionalized after their synthesis showed the best performance for the detection of the complementary target using fluorescence.

### 3.3. Surface Micropattern Fabrication and Characterization

**Figure 4:**
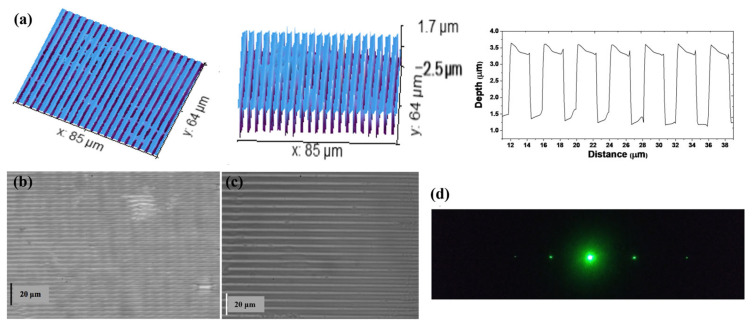
(**a**) Images and cross section profile of the microstructure PET (polyethylene terephthalate) master fabricated with Direct Laser Interference Patterning obtained using a 3D Optical Profilometer (Sensofar, Spain). Optical microscopy image of (**b**) the negative micropattern copied in PDMS using thermal curing and (**c**) the Surface Relief Grating (SRG) replicated in (AM(25)/PA) hydrogel from the PDMS micropattern. (**d**) Optical diffraction observed for the SRG, obtained in (**c**), measured with green laser irradiation (λ = 532 nm) after complete swelling in distilled water.

For the surface micropatterning of hydrogels, PET masters were used to obtain a negative in PDMS which was in turn replicated with the above optimized hydrogel compositions. The fabricated PET master was characterized using confocal microscopy (Figure 4a). The profile obtained from the confocal images shows that the gratings have a period of 4 µm and a depth of 2.1 µm. The PDMS negative copy was characterized with optical microscopy where, as expected, a period of 4 µm was observed, which confirmed the correct replica of the PET master (Figure 4b). In addition, the original PET master and its PDMS copies were irradiated with a continuous green laser at 532 nm using the optical set-up described in the Materials and Methods section (Figure 1), and the diffraction efficiency (DE%) was calculated using Equation (2). Both fabricated microstructures showed good diffraction efficiency.

Hydrogel surface micropatterning was realized, during the polymerization, for the optimized compositions using replica molding. The thermally activated curing process, for Acrylamide/Propargyl acrylate hydrogels, took place in 1.30 h, supposedly a sufficient time for obtaining a good copy of the original PET microstructure. For the AM(25)/PA and AM(25) compositions, a good copy of the microstructure was obtained during the thermal curing. Figure 4c shows the optical microscopy image of the AM(25)/PA hydrogel grating which correctly replicated the pattern. It should be noticed that a higher period is observed in the hydrogel compared to the PDMS master, as the first one is swollen in water. The diffraction of the AM(25)/PA hydrogel, thermally polymerized, was also evaluated after its irradiation with a continuous green laser at 532 nm using the optical set-up of Figure 1. Figure 4d shows the diffraction pattern of the AM(25)/PA hydrogel. Zero, first, and second diffractive orders are present and distinguishable, so it could be very useful for label-free biosensing based on diffractive measurements. The diffraction efficiency (DE%) was calculated for the first diffraction order using Equation (2), resulting in 4.6 ± 0.5, 9.8 ± 0.5, and 1.1 ± 0.2, for PET, PDMS, and AM(25)/PA gratings, respectively. Lower values were observed in comparison with the PET and PDMS master, which was expected as the hydrogel has a watery nature and the PET and PDMS are plastics. The replica of the microstructure using thermal activation was not possible for the AM(8)/PA and AM(8) compositions. This was attributed to the amount of monomer used, which was too low to achieve the right viscosity for the replication process. On the other hand, trials of the grating replica molding using photochemical polymerization resulted unsuccessful, since the polymerization proceeded too fast to permit the correct molding.

On the other hand, by varying UV photoreactor parameters individually for each hydrogel composition, such as UV light power and irradiation time, hydrogel surface micropatterns were successfully obtained for all the optimized hydrogel compositions. However, the peeling-off of the hydrogel surface pattern copied from the PDMS, using photochemical activation, was cumbersome, and thus 20 μL of glycerol was added to promote the detachment. For the (AM(25)/PA), AM(25), and (AM(8)/PA) hydrogels, micro-patterned replicas were obtained using 15 min of UV irradiation and 10 mW/cm^2^ of light power, whereas for the AM(8) hydrogel, 10 min of UV irradiation and 0.6 mW/cm^2^ of light power were used.

Although it was possible to replicate the grating using both thermal and photochemical activation, it was concluded that better reproducibility in surface micropatterns copies was obtained for the AM(25)/PA hydrogel composition during thermal curing. Thus, the AM(25)/PA hydrogel composition showed the best results in terms of micropattern fabrication and biorecognition properties. Consequently, it was chosen for further label-free biosensing studies.

### 3.4. Label-Free Biorecognition

**Figure 5:**
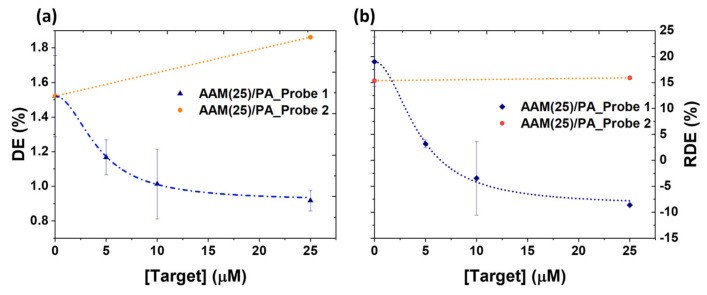
Change in the diffraction efficiency of SRG made of probe-functionalized hydrogels after hybridization with Target 1. (**a**) Diffraction efficiency (DE) measured at λ = 532 nm and, (**b**) relative diffraction efficiency (RDE) of SRG functionalized with Probe 1 (blue) and Probe 2 (orange) hybridized with increasing concentrations of Target 1 (complementary to Probe 1). The DE changes with the amount of Target hybridized only for the SRG hydrogels functionalized with the complementary strand.

To evaluate the potential label-free sensing of surface relief gratings of the probe-functionalized hydrogels, a hybridization assay was performed using unlabeled probes. Firstly, surface microstructures were obtained for the AM(25)/PA hydrogels during the thermal curing as, according to previous results, this hydrogel composition and reaction conditions produced the hydrogel with the best properties for the selective detection of targets with fluorescent sensing, and, in addition, they yielded micropatterned hydrogels that were able to correctly diffract the light. Therefore, the same conditions were expected to produce hydrogels with the best properties for the label-free detection of targets. After the hydrogel synthesis, the AM(25)/PA hydrogel was functionalized with 5 μM of Probe 1. The functionalized hydrogel patterns were placed in a Petri dish and washed overnight with SSC1x buffer. The day after, they were cut into squares (0.5 × 0.5cm) and positioned in separated wells of a transparent ELISA plate with 250 µL of SSC1x. The size of the hydrogel was chosen to perfectly fit within the ELISA wells and, thus, avoid the crushing of their walls and their free flotation. Diffraction efficiencies (DE%) of the functionalized hydrogel patterns were registered using the optical set-up (Figure 1) at controlled conditions (RH 45 ± 5% and 24 ± 1°C). Ambient conditions were reached with domestic air conditioning and humidifier systems. Figure S12 shows that signals were stable for at least 30 min. Therefore, the signal was not affected by the incidence of the focused laser beam and slight delays in the reading time would not affect the obtained results. After that, the hybridization assay was performed in triplicate. Hydrogels were incubated with a growing concentration of Target 1 (0; 5; 10; and 25 µM) in 50 µL of SSC1x for 1h at 37 °C. After overnight washing with SSC1x, DE was registered at 532 nm and RDE was calculated according to Equation (3) to assess the direct detection of complementary DNA-sequence (Target 1) (Figure 5). As a control experiment, the AM(25)/PA hydrogel was also functionalized with a non-complementary DNA sequence (Probe 2), and hybridization assays were performed with Target 1 at 25 µM following exactly the same procedure. A gradual decrease in the DE% with increasing concentration of the unlabelled target was observed for the (AM(25)/PA) hydrogel functionalized with Probe 1, while for the control system, having immobilized the non-complementary sequence Probe 2, no tendency was observed. The DE (%) data obtained with probe 1 can be best fitted using a Hill 1 correlation curve, obtaining a correlation coefficient of R^2^ = 0.991. The RDE (%) data obtained with Probe 1 can also be best fitted using a Hill 1 correlation curve, obtaining a correlation coefficient of R^2^ = 0.997. The limit of detection (LOD) of 2.47 µM was calculated from the RDE (%) curve as the concentration associated with the mean signal of ten blank measurements plus three times their standard deviation. Thus, it was possible to detect the analyte in the range from 2.47 to 10 μM using the micropatterned hydrogels as an optical transducer.

Therefore, the label-free biosensing assay using unlabeled probes, performed for (AM(25)/PA) hydrogels with the surface micropattern, showed excellent preliminary results. The LOD of DNA in our system is higher lower than most of the hydrogel-based systems described in the literature [40]. However, most of the approaches are based on labels or/and elaborate DNA architectures. DNA hybridization with hydrogel has also been explored for actuators and other purposes [41], but poor consideration of the analytical performance is contemplated in these studies. Baba and co-workers have reported the use of diffraction gratings for the label-free detection of DNA with very low LOD, but the DNA was amplified during the analysis [42]. Our results are very promising, but the diffraction efficiency calculated for the obtained hydrogel surface-micropattern is not high. Hence, further improvements in the micropattern fabrication can be realized to increase the initial DE% and, accordingly, the sensitivity for the analyte detection. These improvements involve the fabrication of thinner surface relief gratings as well as the replication with lower-period PET masters. Although fabrication of these gratings can be challenging, technologies such as two-photon polymerization can be used for fabricating 2D/3D microstructures with high accuracy [43,44]. In addition, quicker data acquisition and automatization of hydrogel SRGs will allow for increasing the number of replicates and lowering the experimental error. Despite those facts, it was possible to directly detect the analyte with good selectivity and sensitivity, given that this is the first time that surface micro-patterned hydrogels were used to directly detect hybridization events.

## 4. Conclusions and Future Outlook

Optical biosensors are emerging for point-of-care testing (POCT) as they present some advantages such as increased sensitivity and suitability for being integrated into a compact device with the purpose of being utilized out-of-the-lab. Overall, line-like periodic microstructures were successfully fabricated on a bioresponsive hydrogel surface and used as transducers for converting the analyte–bioreceptor binding into a measurable optical signal. The planned approach for the covalent immobilization of the bioreceptor probes had notable outcomes. Furthermore, different bioreceptors with thiol terminal groups could be used, depending on the analyte to be detected. Accordingly, the developed biosensor can sense multiple analytes. Results obtained from the label-free biorecognition assay have shown a direct correlation between the diffraction efficiency measured and the target concentration. The label-free biosensor as designed could significantly contribute to direct and accurate analysis in medical diagnosis, being cheap, easy to fabricate and working without the need for further reagents. To fully achieve this, further aspects should be considered, such as the minimization of biofouling of hydrogels when they are immersed in real fluids. This can be achieved by tuning the composition of hydrogels, for instance, using polyacrylamide copolymers or zwitterionic moieties [45].

## Figures and Tables

**Table 1:** Optimized hydrogel compositions.

Hydrogel	AM (% *w*/*v*)	MBA (% *w*/*v*)	PA (μL)	DI water (μL)
AM(25)	25	0.05	0	1000
AM(25)/PA	25	0.05	15	1000
AM(8)	8	0.25	0	1000
AM(8)/PA	8	0.25	15	1000

## Data Availability

The data presented in this study are available on request from the corresponding author. The data are not publicly available due to privacy restrictions.

## Supplementary Materials

The following supporting information can be downloaded at: https://www.mdpi.com/article/10.3390/bios13030312/s1. Table S1. Nucleotide Sequence of Probes and Targets used. Scheme S1. Schematic representation of the hydrogel synthesis by free-radical polymerization (FRP). AM: Acrylamide, MBA: N, N’-methylenebis (acrylamide), PA: propargyl acrylate, Initiator = DMPA: 2,2-Dimethoxy-2-phenylacetophenone. Scheme S2. Thiol probe immobilization by thiolene and thiol-yne click reaction of (AM/PA) hydrogels by UV light. AM: Acrylamide, PA: propargyl acrylate, Initiator = DMPA: 2,2-Dimethoxy-2-phenylacetophenone. Table S2. Hydrogel compositions. Figure S1. UV-Visible spectra of hydrogels with different compositions (a) without PA and (b) with PA. Figure S2. Digital images of hydrogels pieces with different compositions and consistency AM(8)/PA_0.050, soft; (a), (b) AM(8)/PA_0.250, adaptable and (c) AM(32)/PA_0.250, brittle. Figure S3. Digital images of selected hydrogels pieces with different compositions (a) AM(8)_0.250, (b) AM(25)/PA_0.050 (c) AM(8)/PA_0.250, and (d) AM(25)_0.250. Figure S4. Porosity observed by SEM for selected hydrogel compositions (AM(25)/PA_0.050) and (AM(8)/PA_0.250) prepared by thermal and photochemical activation. Figure S5. Swelling kinetic studies for (AM(25)/PA), (AM(8)/PA), AM(25) and AM(8) hydrogels soaked in PSB-T (obtained by thermal activation). Figure S6. ATR-FTIR spectrum of AM(25) hydrogel. Figure S7. Fluorescence signals obtained for probe-functionalized a)(AM(8)/PA) hydrogel and b) (AM(25)/PA) after hybridization with Target 2 for 1h at 37 °C (λex = 633 nm, λem = 670 nm). Firstly, hydrogels of were functionalized during the synthesis, using the first strategy (one-pot, photochemical) with 1 µM of the thiolated probes: Probe 1. After overnight washing with PBS-T, they were hybridized with 1 µM of fluorescent-labeled Target 2. Hydrogels were cutted in three pieces and the central piece was flipped prior to analysis to observe the signals of the cross-section profile. Fluorescence signals were collected after hybridization. Experiment was carried out in triplicate (three rows of the images). The fluorescence signal is visible in all thee pieces for both hydrogels. Figure S8. Fluorescence signals obtained for probe-functionalized (AM(25)/PA) hydrogel after hybridization with Target 2 (λex = 633 nm, λem = 670 nm). Firstly, hydrogels were functionalized during the synthesis, using the first strategy (one-pot, photochemical) with 1 µM of the thiolated probes: Probe 1 and, as a control, Probe 2. After overnight washing with PBS-T, they were hybridized with 1 µM of fluorescent-labeled Target 2. Fluorescence signals were collected after hybridization and 2 hours washing and after overnight washing with SSC1x. The fluorescence signal remained only in the case of Probe 1, complementary to the Target. Figure S9. Fluorescence signals obtained for AM(25) and (AM(25)/PA) hydrogels through hybridization assay with Target 2 (λex = 633 nm, λem = 670 nm). Firstly, hydrogels were biofunctionalized with thiolated probes (Probe 1 and Probe 2) at 1 µM after the polymerization. In the first bar chart, fluorescence signals were registered just after the hybridization assay with 0.5 µM of Target 2. In the second bar chart, the fluorescence was registered after overnight washing with SSC1x in order to wash away all the non-specific binding. Figure S10. Fluorescence signals obtained for (AM(8)/PA) hydrogel through hybridization assay with Target 2 (λex = 633 nm, λem = 670 nm). Firstly, hydrogels were functionalized with thiolated probes (Probe 1 and the control probe Probe 2) at 1 µM during the synthesis, using the one-pot synthesis strategy. After overnight washing with PBS-T, they were hybridized with 1 µM of the Target 2. Fluorescence signals, after hybridization, were collected after overnight washing with SSC1x. The experiment was conducted in triplicate. Figure S11. Fluorescence signals obtained for AM(8) and (AM(8)/PA) hydrogels through hybridization assay with Target 2 (λex = 633 nm, λem = 670 nm). Firstly, hydrogels were functionalized with thiolated probes (Probe 1 and, as control probe, Probe 2) at 1 µM after the synthesis, using the two-step strategy. In the first bar chart, fluorescence signals were registered just after the hybridization assay with 1 µM of the Target 2. In the second bar chart, the fluorescence was registered after overnight washing with SSC 1x in order to wash away all the non-covalent probe binding. Figure S12. Stability of the measured signals with the optical setup over night: Intensities of the zero and first diffraction orders generated by the AM(25)/PA hydrogel immersed in SSC1X withing the wells of the plate were registered with the photodiodes after illumination with the laser beam (λ = 532 nm).
