# Supplementary material for: Surface Micro-Patterned Biofunctionalized Hydrogel for Direct Nucleic Acid Hybridization Detection
Source: Biosensors (Basel). 2023 Feb 23;13(3):312. doi: 10.3390/bios13030312 (PMC10046352; doi:10.3390/bios13030312)
Supplement: Supplementary file 1 [file biosensors-13-00312-s001.zip › biosensors-2219219-supplementary.pdf]

*Supplementary Material*

# Surface Micro-Patterned Biofunctionalized Hydrogel for Direct Nucleic Acid Hybridization Detection

**Paola Zezza [1], María Isabel Lucío [1], Estrella Fernández [1], Ángel Maquieira [1,2] and María-José Bañuls [1,2,*]**

## S-I Nucleotide Sequence of Probes and Targets Used

**Table S1.** Nucleotide Sequence of Probes and Targets used.

| Name | Sequence (5′ to 3′) | 5′ end | 3′end |
|---|---|---|---|
| Probe 1 | CCCGATTGACCAGCTAGCATT | SH | None |
| Probe 2 (control probe) | ATCGACACCCCTATCACGATT | SH | None |
| Target 1 | AATGCTAGCTGGTCAATCGGG | None | None |
| Target 2 | AATGCTAGCTGGTCAATCGGG | Cy5 | None |

## S-II Scheme of Hydrogel Synthesis

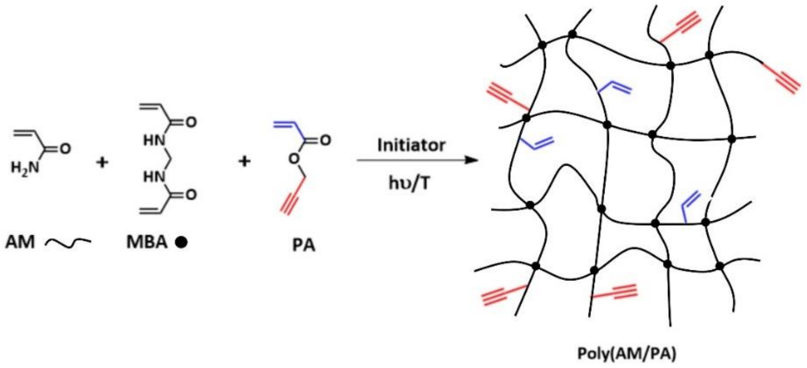


**Scheme S1.** Schematic representation of the hydrogel synthesis by free-radical polymerization (FRP). AM: Acrylamide, MBA: N, N′-methylenebis (acrylamide), PA: propargyl acrylate, Initiator=DMPA: 2,2-Dimethoxy-2-phenylacetophenone.

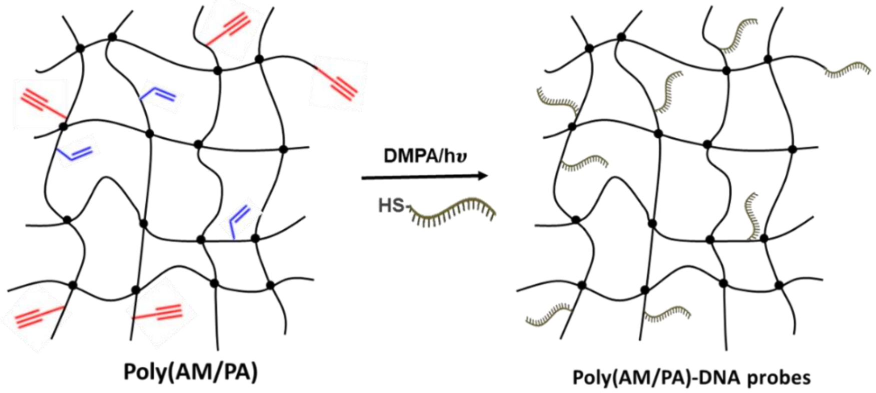


**Scheme S2.** Thiol probe immobilization by thiol-ene and thiol-yne click reaction of (AM/PA) hydrogels by UV light. AM: Acrylamide, PA: propargyl acrylate, Initiator=DMPA: 2,2-Dimethoxy-2-phenylacetophenone.

## S-III Hydrogel Composition Optimization

**Table S2.** Hydrogel compositions.

| Entry | Hydrogel | AM (% *w/v*) | MBA (% *w/v*) | PA (μL) | Consistency / Appearance |
|---|---|---|---|---|---|
| 1 | AM(8)_0.050 | 8 | 0.050 | 0 | Soft |
| 2 | AM(8)_0.125 | 8 | 0.125 | 0 | Soft |
| 3 | AM(8)_0.250=AM(8) | 8 | 0.250 | 0 | Adaptable |
| 4 | AM(8)/PA_0.050=AM(8)/PA | 8 | 0.050 | 15 | Soft |
| 5 | AM(8)/PA_0.125 | 8 | 0.050 | 15 | Soft |
| 6 | AM(8)/PA_0.250 | 8 | 0.125 | 15 | Adaptable |
| 7 | AM(25)_0.050=AM(25) | 25 | 0.050 | 0 | Adaptable |
| 8 | AM(25)_0.125 | 25 | 0.125 | 0 | Adaptable |
| 9 | AM(25)_0.250 | 25 | 0.250 | 0 | Brittle |
| 10 | AM(25)/PA_0.050=AM(25)/PA | 25 | 0.050 | 15 | Adaptable |
| 11 | AM(25)/PA_0.125 | 25 | 0.125 | 15 | Brittle |
| 12 | AM(25)/PA_0.250 | 25 | 0.250 | 15 | Brittle |
| 13 | AM(32)_0.050 | 32 | 0.050 | 0 | Adaptable |
| 14 | AM(32)_0.125 | 32 | 0.125 | 0 | Brittle |
| 15 | AM(32)_0.250 | 32 | 0.250 | 0 | Brittle |
| 16 | AM(32)/PA_0.050 | 32 | 0.050 | 15 | Brittle |
| 17 | AM(8)/PA_0.125 | 32 | 0.125 | 15 | Brittle |
| 18 | AM(32)/PA_0.250 | 32 | 0.250 | 15 | Brittle |

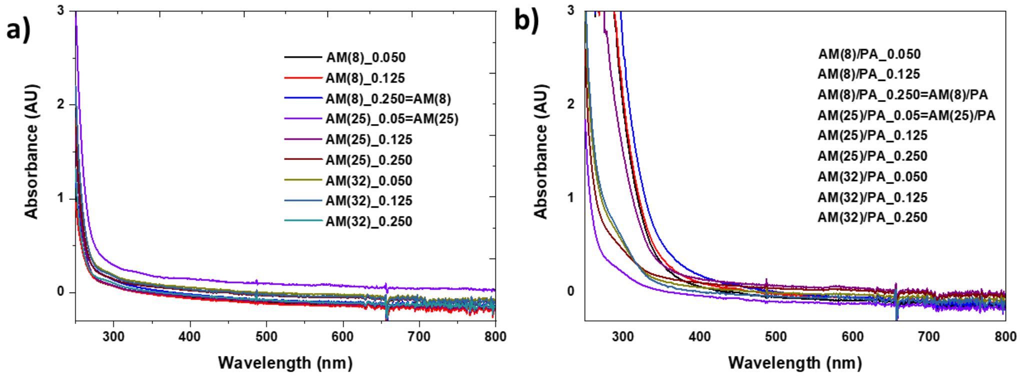


**Figure S1.** UV-Visible spectra of hydrogels with different compositions a) without PA and b) with PA.

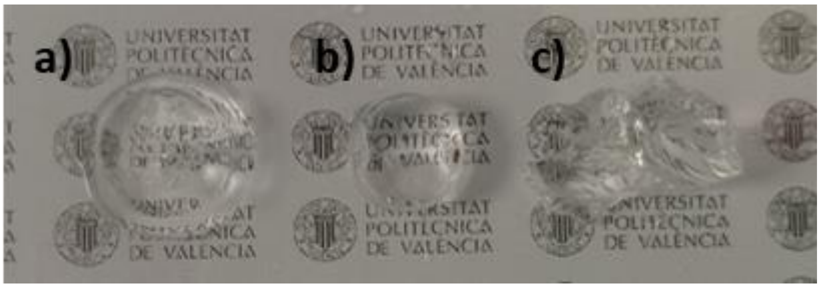


**Figure S2.** Digital images of hydrogels pieces with different compositions and consistency AM(8)/PA_0.050, soft; b), b) AM(8)/PA_0.250, adaptable and c) AM(32)/PA_0.250, brittle.

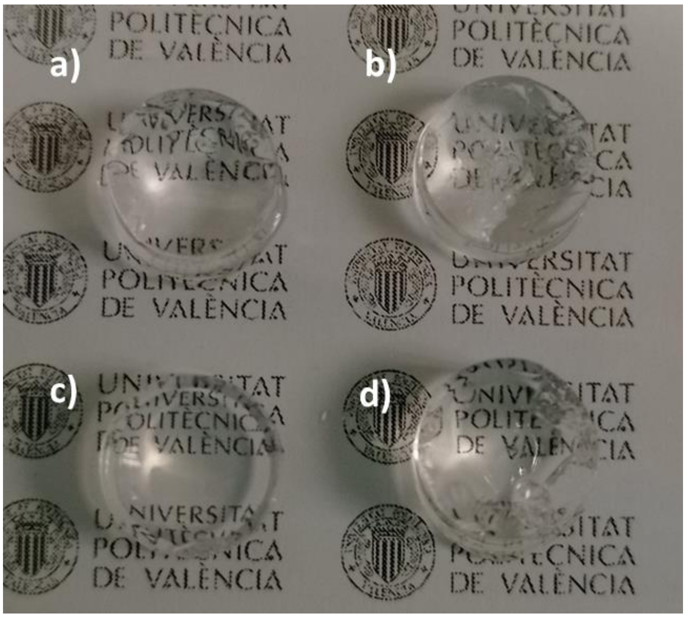


**Figure S3.** Digital images of selected hydrogels pieces with different compositions a) AM(8)_0.250, b) AM(25)/PA_0.050 c) AM(8)/PA_0.250, and d) AM(25)_0.250.

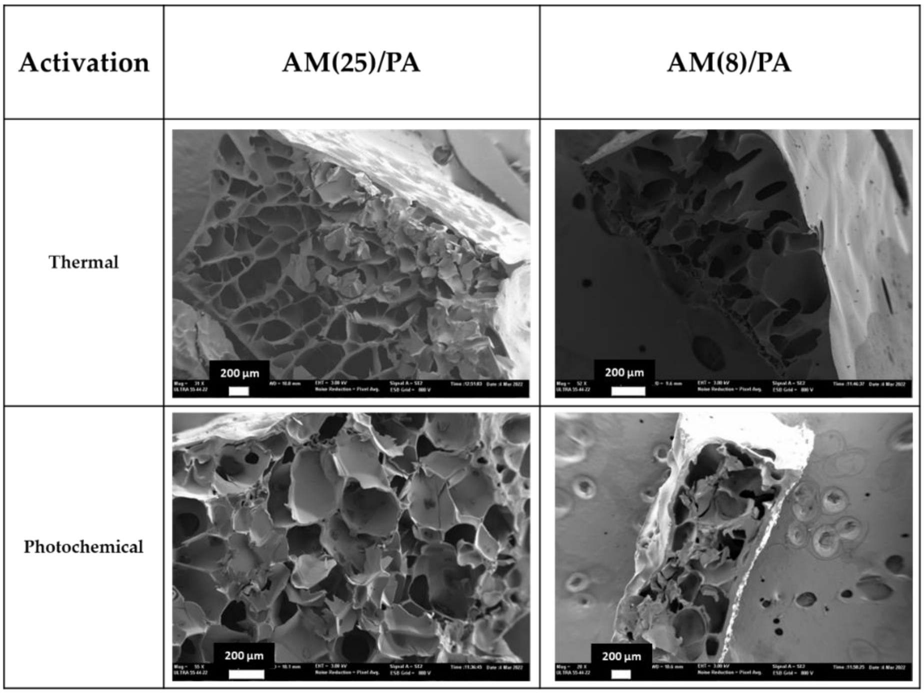


**Figure S4.** Porosity observed by SEM for selected hydrogel compositions (AM(25)/PA_0.050) and (AM(8)/PA_0.250) prepared by thermal and photochemical activation.

## S-IV Swelling Degree of Hydrogel

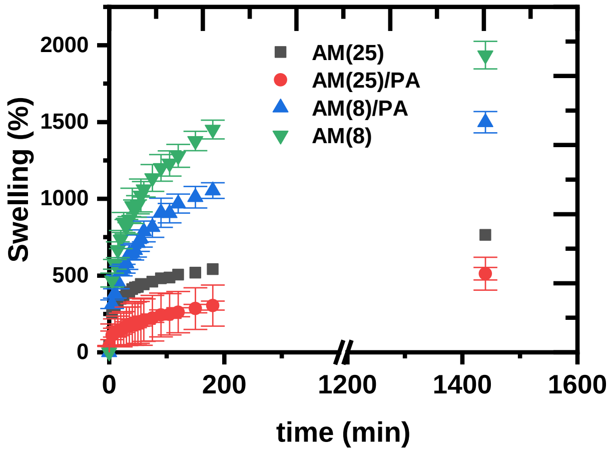


**Figure S5.** Swelling kinetic studies for (AM(25)/PA), (AM(8)/PA), AM(25) and AM(8) hydrogels soaked in PSB-T (obtained by thermal activation).

## S-V ATR-FTIR Spectrum of AM(25) Hydrogel

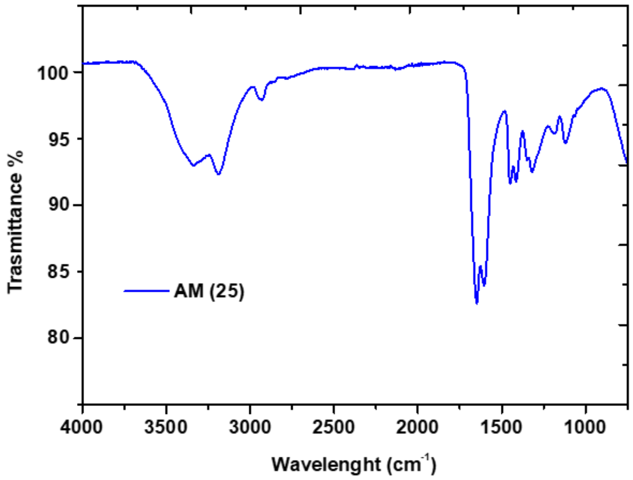


**Figure S6.** ATR-FTIR spectrum of AM(25) hydrogel.

## S-VI Details of Fluorescence Signals

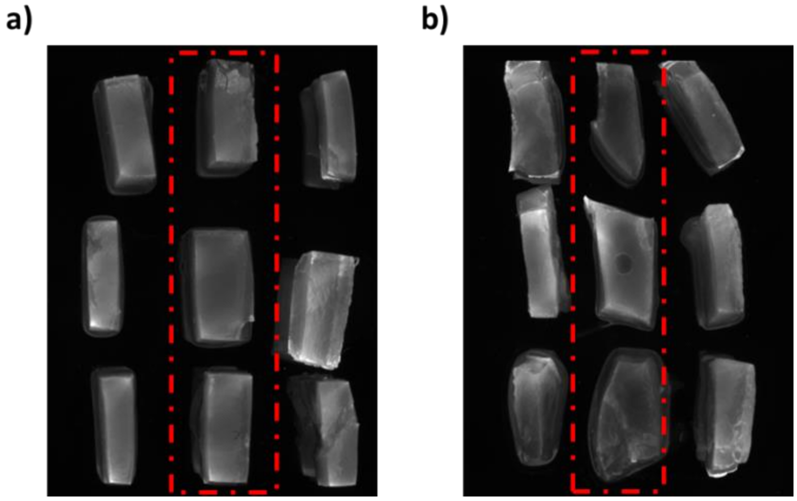


**Figure S7.** Fluorescence signals obtained for probe-functionalized a)(AM(8)/PA) hydrogel and b) (AM(25)/PA) after hybridization with Target 2 for 1h at 37°C ($\lambda$ex = 633 nm, $\lambda$em = 670 nm). Firstly, hydrogels of were functionalized during the synthesis, using the first strategy (one-pot, photochemical) with 1 μM of the thiolated probes: Probe 1. After overnight washing with PBS-T, they were hybridized with 1 μM of fluorescent-labeled Target 2.  Hydrogels were cutted in three pieces and the central piece was flipped prior to analysis to observe the signals of the cross-section profile. Fluorescence signals were collected after hybridization. Experiment was carried out in triplicate (three rows of the images). The fluorescence signal is visible in all thee pieces for both hydrogels.

| Label | (AM(25)/PA) + Probe 2 | (AM(25)/PA) + Probe 1 | AM(25) + Probe 1 |
|---|---|---|---|
| After 2 hours washing | 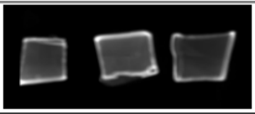 | 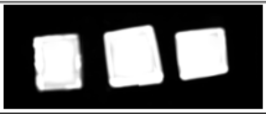 | 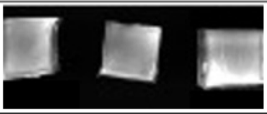 |
| After overnight washing | 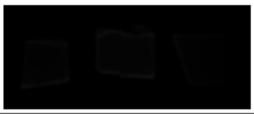 | 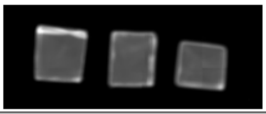 | 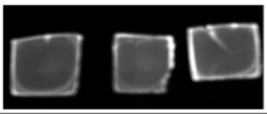 |

**Figure S8.** Fluorescence signals obtained for probe-functionalized (AM(25)/PA) hydrogel after hybridization with Target 2 ($\lambda$ex = 633 nm, $\lambda$em = 670 nm). Firstly, hydrogels were functionalized during the synthesis, using the first strategy (one-pot, photochemical) with 1 μM of the thiolated probes: Probe 1 and, as a control, Probe 2. After overnight washing with PBS-T, they were hybridized with 1 μM of fluorescent-labeled Target 2. Fluorescence signals were collected after hybridization and 2 hours washing and after overnight washing with SSC1x. The fluorescence signal remained only in the case of Probe 1, complementary to the Target.

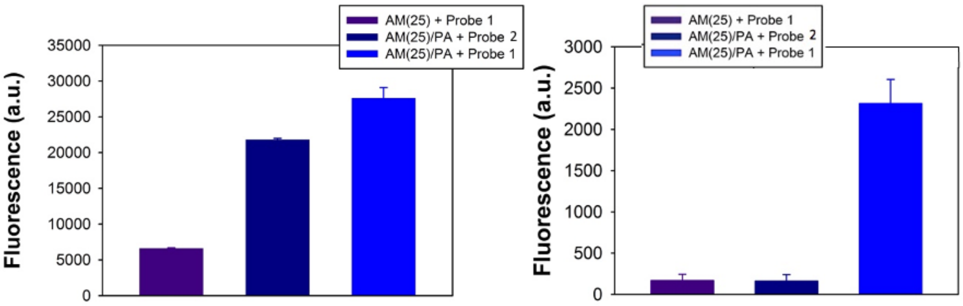


| Label | AM(25) + Probe 1 | AM(25)/PA + Probe 2 | AM(25)/PA + Probe 1 |
|---|---|---|---|
| Before washing | 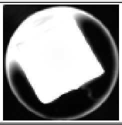 | 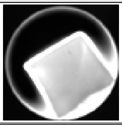 | 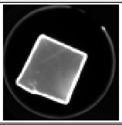 |
| After overnight washing | 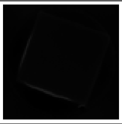 | 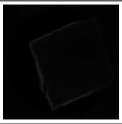 | 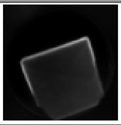 |

**Figure S9.** Fluorescence signals obtained for AM(25) and (AM(25)/PA) hydrogels through hybridization assay with Target 2 ($\lambda$ex = 633 nm, $\lambda$em = 670 nm) . Firstly, hydrogels were biofunctionalized with thiolated probes (Probe 1 and Probe 2) at 1 µM after the polymerization. In the first bar chart, fluorescence signals were registered just after the hybridization assay with 0.5 µM of Target 2. In the second bar chart, the fluorescence was registered after overnight washing with SSC1x in order to wash away all the non-specific binding.

| Label | AM(8) + Probe 1 | AM(8)/PA + Probe 2 | AM(8)/PA + Probe 1 |
|---|---|---|---|
| Before washing | 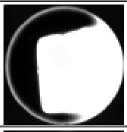 | 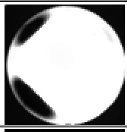 | 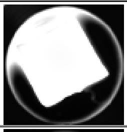 |
| After overnight washing | 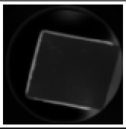 | 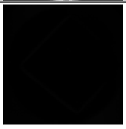 | 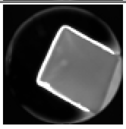 |

**Figure S10.** Fluorescence signals obtained for (AM(8)/PA) hydrogel through hybridization assay with Target 2 ($\lambda$ex = 633 nm, $\lambda$em = 670 nm). Firstly, hydrogels were functionalized with thiolated probes (Probe 1 and the control probe Probe 2) at 1 µM during the synthesis, using the one-pot synthesis strategy. After overnight washing with PBS-T, they were hybridized with 1 µM of the Target 2. Fluorescence signals, after hybridization, were collected after overnight washing with SSC1x. The experiment was conducted in triplicate.

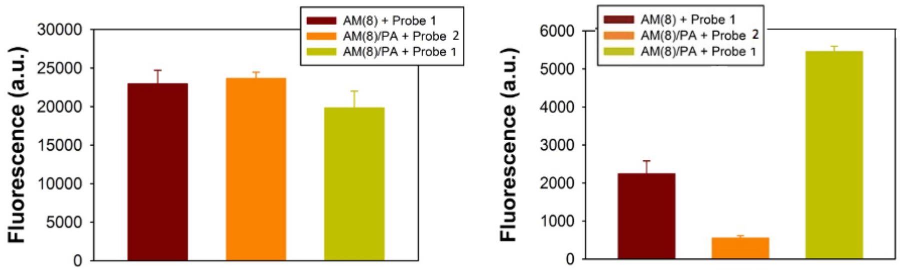


| Label | AM(8) + Probe 1 | AM(8)/PA + Probe 2 | AM(8)/PA + Probe 1 |
|---|---|---|---|
| Before washing | 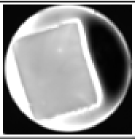 | 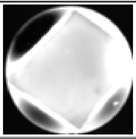 | 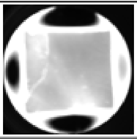 |
| After overnight washing | 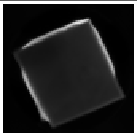 | 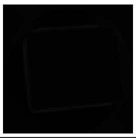 | 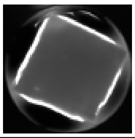 |

**Figure S11.** Fluorescence signals obtained for AM(8) and (AM(8)/PA) hydrogels through hybridization assay with Target 2 ($\lambda$ex = 633 nm, $\lambda$em = 670 nm). Firstly, hydrogels were functionalized with thiolated probes (Probe 1 and, as control probe, Probe 2) at 1 µM after the synthesis, using the two-step strategy. In the first bar chart, fluorescence signals were registered just after the hybridization assay with 1 µM of the Target 2. In the second bar chart, the fluorescence was registered after overnight washing with SSC 1x in order to wash away all the non-covalent probe binding.

## S-VII Diffraction Efficiency Measurements

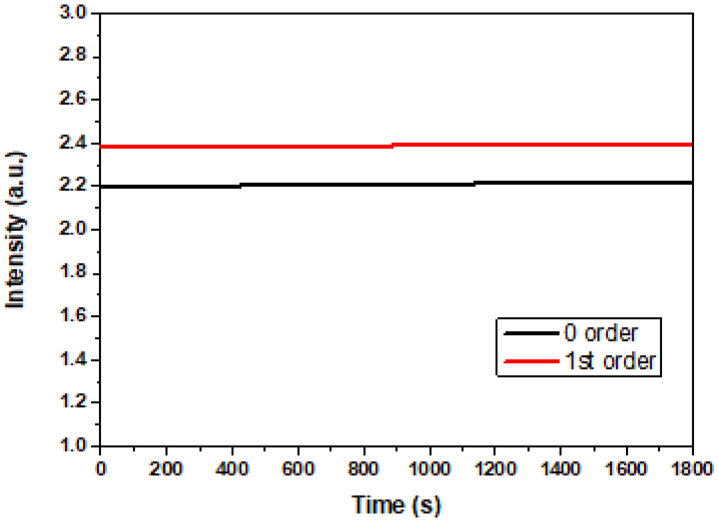


**Figure S12.** Stability of the measured signals with the optical setup over night: Intensities of the zero and first diffraction orders generated by the AM(25)/PA hydrogel immersed in SSC1X withing the wells of the plate were registered with the photodiodes after illumination with the laser beam ($\lambda$=532 nm).
